# Author Correction: Impacts of environmental and socio-economic factors on emergence and epidemic potential of Ebola in Africa

**DOI:** 10.1038/s41467-019-12967-z

**Published:** 2019-11-15

**Authors:** David W. Redding, Peter M. Atkinson, Andrew A. Cunningham, Gianni Lo Iacono, Lina M. Moses, James L. N. Wood, Kate E. Jones

**Affiliations:** 1 Centre for Biodiversity and Environment Research, Department of Genetics, Evolution and Environment, University College London, Gower Street, London, WC1E 6BT UK; 2 Lancaster Environment Centre, Lancaster University, Bailrigg Lancaster, LA4 1YW UK; 3 Institute of Zoology, Zoological Society of London, Regent’s Park, London, NW1 4RY UK; 4 School of Veterinary Medicine, University of Surrey, Guildford, UK; 5 Department of Global Community Health and Behavioral Sciences, Tulane University, New Orleans, LA USA; 6 Department of Veterinary Medicine, Disease Dynamics Unit, University of Cambridge, Cambridge, UK

**Keywords:** Climate-change ecology, Macroecology, Ecological epidemiology, Infectious diseases

Correction to: *Nature Communications* 10.1038/s41467-019-12499-6, published online 15 October 2019.

The original version of the published article had an error in the abstract, which originally stated “yield a fourfold higher likelihood of epidemics”. This has been corrected to “yield a 1.63-fold higher likelihood of epidemics”.

