# Supplementary material for: Impacts of environmental and socio-economic factors on emergence and epidemic potential of Ebola in Africa
Source: Nat Commun. 2019 Oct 15;10:4531. doi: 10.1038/s41467-019-12499-6 (PMC6794280; doi:10.1038/s41467-019-12499-6)
Supplement: Supplementary file 1 — Supplementary Information [file 41467_2019_12499_MOESM1_ESM.pdf]

Supplementary Methods

<u>EMM compartmental transition algorithm.</u>

For each time step t, the number of individuals moving through disease compartments both within and between grid cells (see Fig. 2) was estimated using disease transmission parameters. We predicted the likely movement between disease compartments per time step, by drawing randomly from a binomial distribution. We describe this process below, using as an example the movement of individuals moving from Exposed to Infectious compartments within grid cells.

1. We determined the probability that a number of individuals were likely to move from the Exposed to Infectious compartments as:

$$p(k_i \text{ infections within } E_t) = \binom{E_t}{k_i} \alpha_t^{k_i}(1-\alpha_t)^{E_t-k_i} \quad \text{(Equation 1)}$$

   where k_i represents the number of individuals that enter the Infectious compartment, E_t the number of Exposed individuals at time t, and α_t the transition probability at time t.
2. Using Equation 1, we determined for any value of k_i the probability of k_i individuals that move into the Infectious compartment, i.e., we computed the probability of the number of people, between 0 and the total number of individuals in the Exposed compartment, entering the Infectious compartment at time step t. We then drew randomly from this probability distribution to choose k_i individuals that moved into the Infectious compartment, thereby weighting the choice towards the more likely outcomes given α.
3. Once the number of people that will be infected in the next time step k_i was determined, then k_i individuals were removed from the Exposed compartment and added to the Infectious compartment.
4. This process continued (per time step) until the number of individuals in the Exposed compartment equalled zero.

The same process was applied to every compartment change using the respective transition probabilities (i.e., substituting α in the above example). Movement of individuals between respective Exposed and Infectious compartments between grid cells was also modelled similarly, but stopping movements if the exposed or infectious number dropped to zero but with no change to susceptible numbers. Due to the high morbidity from this disease, individuals in the Infectious compartment were deemed less likely to travel and were awarded a travel probability that was half of the expected rate for non-symptomatic individuals.

Force of zoonotic infection, λ_z algorithms.

The force of infection for zoonotic host to human transmission, λ_z was estimated per grid cell, per time step *t,* as follows:

$$\lambda z_t = \kappa H \qquad \text{(Equation 2)}$$

where κ = spill-over risk, and H = probability of zoonotic host presence per grid cell. Spill-over event probability, κ per person, per time step is given by:

$$\kappa = \left(\frac{O}{S_h T}\right) \qquad \text{(Equation 3)}$$

where O = number of historic outbreaks, S_h = number of historically susceptible individuals and T = total time when infections could have occurred. Note: Above we are estimating the probability of an individual being involved in a spill-over event directly from an animal host, which is distinct from the overall risk of contracting the disease.

Force of infection, λ algorithms.

The force of infection for human-to-human transmission, per grid cell and per time step *t,* was estimated as:

$$\lambda_t = \beta I_t + \beta F_t \qquad \text{(Equation 4)}$$

where β = effective contact rate, I_t = number of individuals in Infectious compartment at time step *t,* and F_t = number of individuals in Funeral compartment at time step *t*. For simplicity we assumed that βI and βF were the same (hereafter referred to as β). When $t = 0$, β is given by:

$$\beta = m * \left(\frac{R_0}{ND}\right) \qquad \text{(Equation 5)}$$

where R_0 = basic reproduction number, m = mobility, N = population size per time step, and D = duration in days that an individual is infectious. In this context, m was used to modify the ideal free gas model of human movement with distances travelled which are spatially variable across the landscape. We calculated a two-dimensional collision frequency c, per person per grid cell (1) as follows:

$$c = \; nv\Delta tq2 \qquad \text{(Equation 6)}$$

where n = number of individuals, v = walking velocity, Δt = time period and q = interaction sphere radius. In the context of our simulation, $v\Delta t$ represents daily walking distance. Then we defined m as

the inverse deviation from a mean of c such that areas with more movement have a higher effective contact rate. However, when $t > 0$ we redefined β as follows:

$$\beta = m * \left(\frac{R_e}{ND}\right) \qquad \text{(Equation 7)}$$

where R_e = effective reproduction number, m = mobility, N = population size per time step, and D = duration in days that an individual is infectious. R_e is related to R_0 but due to changes in human behaviour and health care responses, R_e may be lower and decline over time, in addition to the implicit reduction in R as the pool of susceptibles decreases during an outbreak. We assume that the effective reproduction number reduces on a daily basis due to increasingly strong health care responses over time.

So initially, when t = 1:

$$R_e = aR_0 \qquad \text{(Equation 8)}$$

where R_e = effective reproduction number at t = 1, a = decay rate, and R_0 = basic reproduction number. However, when t >1:

$$R_e^{t+1} = aR_e^t \qquad \text{(Equation 9)}$$

where R_e^t= effective reproduction number at time t, and a = decay rate. We define decay rate a per grid cell, from the empirical relationship between wealth and health outcomes. Using either direct or derived empirical estimates of the gradient of the change in R_e over time from (3, 4, 5, 6), we fitted an exponential decay curve between estimates of per captia Gross Domestic Product measured as Purchasing Power Parity (from 2) and the gradient of R_e change per day. The starting R_e decay value a per grid cell, was given by:

$$a = 1.024\ x\ \text{GDP}^{-w2} \qquad \text{(Equation 10)}$$

where the best estimate for exponent w2 was -0.848, GDP = Gross Domestic Product from (7), pseudo r^2 = 0.76, and n = 8.

The poverty-weighted Case Fatality Rate (wCFR) per grid cell, was given by:

$$\text{wCFR} = 0.21\ \ln\left(\frac{1}{\text{GDP}}\right)^{-w1} \qquad \text{(Equation 11)}$$

where the best estimate for exponent w1 was -0.0239, GDP = Gross Domestic Product from (7), pseudo r^2 = 0.9081, and n = 20.

Supplementary Figures

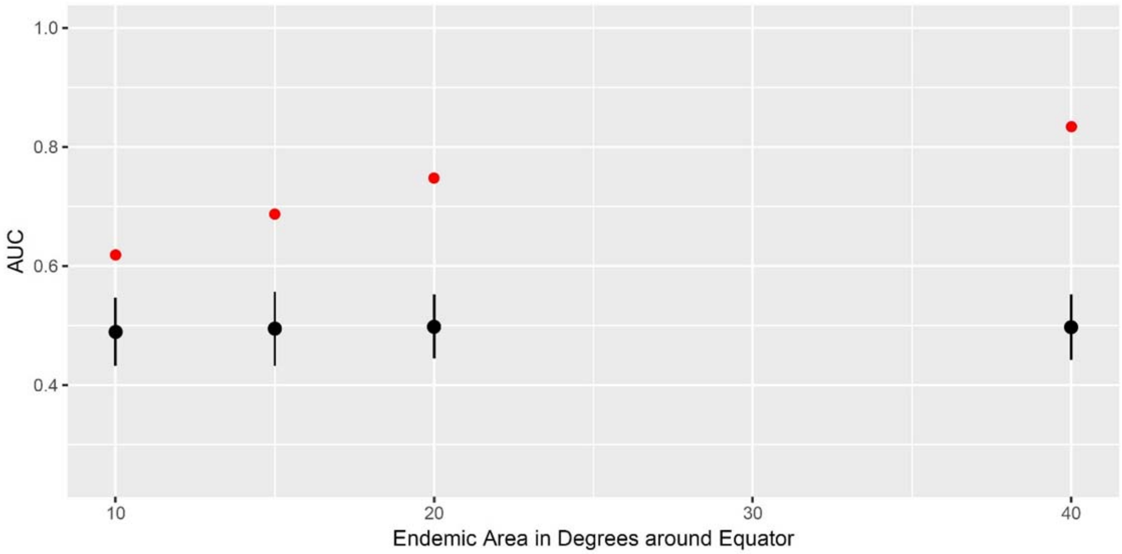

Supplementary Figure 1. **Comparison of AUC scores for predicted risk versus observed risk of known Ebola outbreaks.** Black dots represent four different lots of ten thousand randomisations of the risk surface, whiskers 95% confidence intervals, while red dots are AUC values on the raw predictive surface. The x-axis represents the predicted size of the endemic areas with randomisations of the risk surface happening 10-40 degrees around the equator.

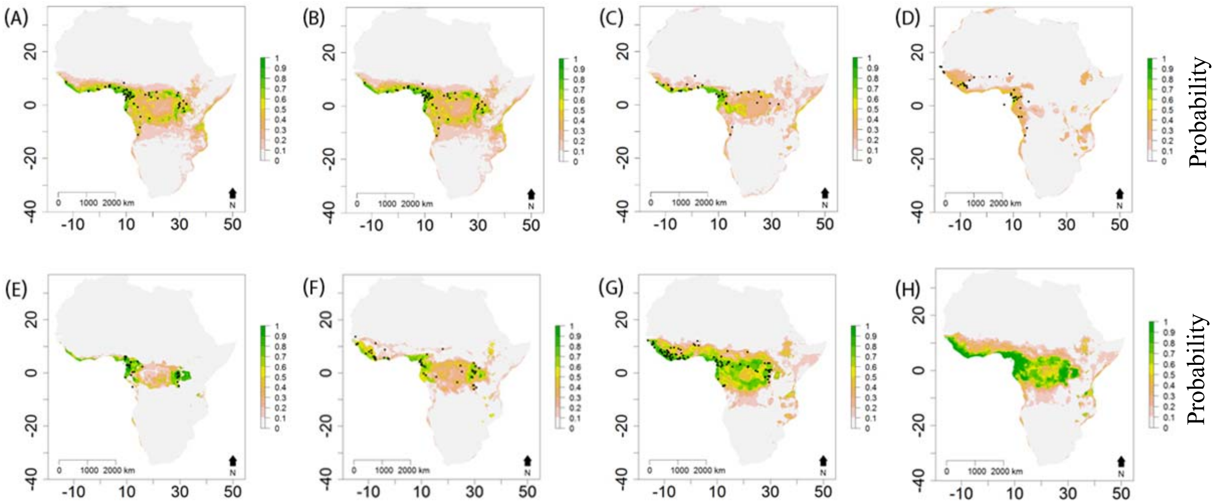

Supplementary Figure 2. **Present-day host occurrence probability.** Maps represent occurrence probability *H* of EBOV host and other infection source species estimated from boosted-regression trees (BRT) models. Probability of species occurrence per grid cell (0.0416°) is represented on a linear color scale where green is most suitable (p(*H*) = 1) and white unsuitable (p(*H*) = 0) where (A) *Epomophorus gambianus gambianus*; (B) *Epomops franqueti*; (C) *Hypsignathus monstrosus*; (D) *Rousettus aegyptiacus*; (E) *Gorilla spp*.; (F) *Pan spp*.; (G) *Cephalophus spp*.; and (H) all species combined. Axis labels indicate degrees in a World Geodetic System 84 projection. Filled black circles represent GBIF (8) occurrence records.

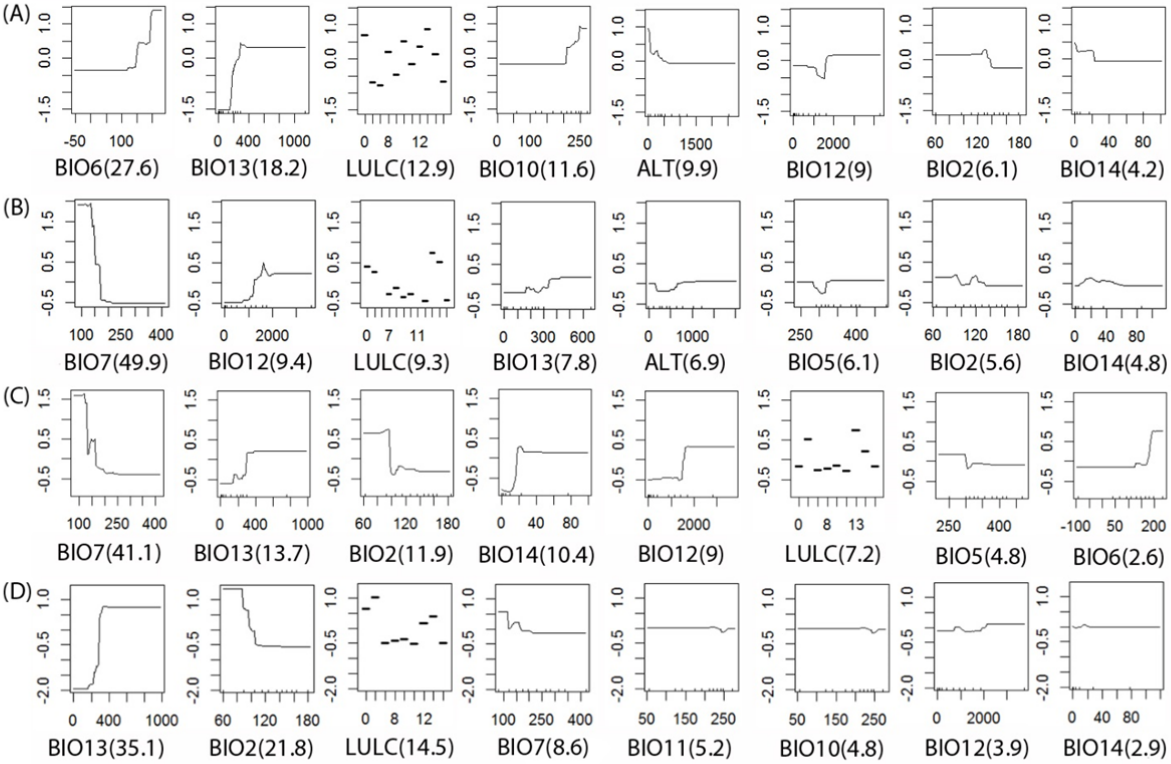

Supplementary Figure 3. Response curves from boosted-regression trees (BRT) models of EBOV host species occurrences. Each plot represents the shape of the normalized fitted functions for each variable where (A) *Epomophorus gambianus gambianus*; (B) *Epomops franqueti*; (C) *Hypsignathus monstrosus*; and (D) *Rousettus aegyptiacus*. The relative percentage contribution of each variable to the model in terms of variance explained is given in parenthesis, where only the top eight variables of the model are included for each species. Variable abbreviations are defined in Supplementary Table 2.

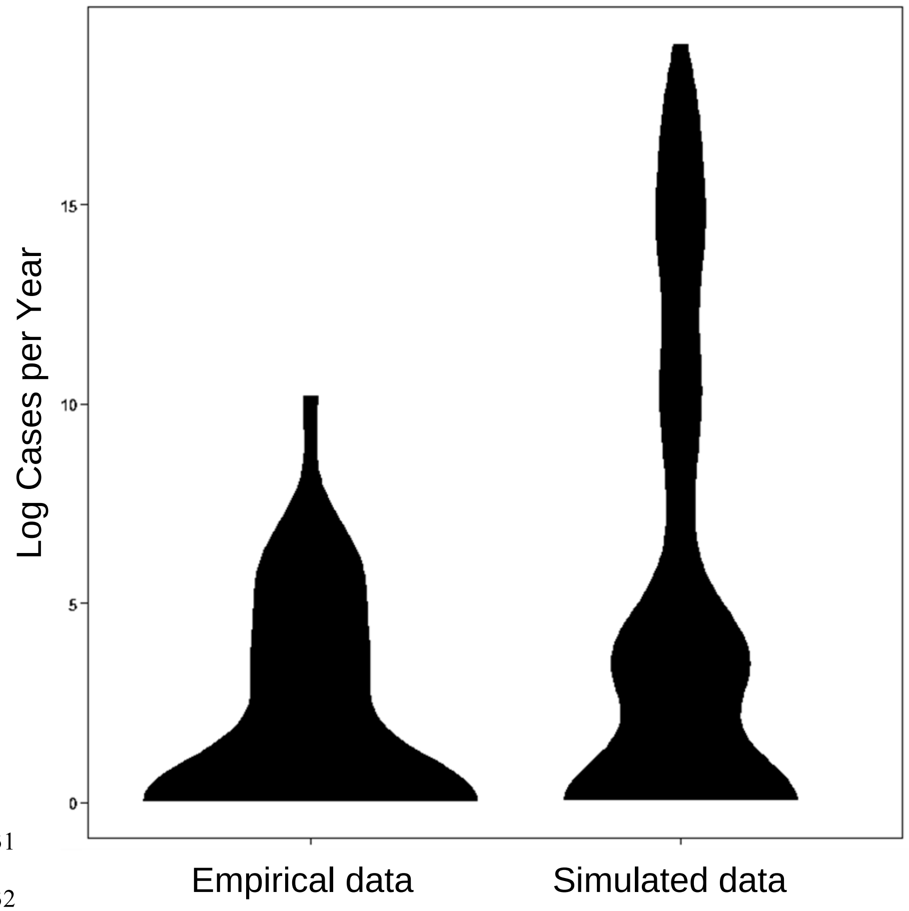

Supplementary Figure 4. Distributions of relative frequency of EBOV cases per year. Violin plots represent the empirical observed (n=23 outbreaks) data of log total number of cases per year from 1967-2016 (9), and log total number of cases per year (n=2500 runs) from EMM simulations for present day environmental and demographic conditions.

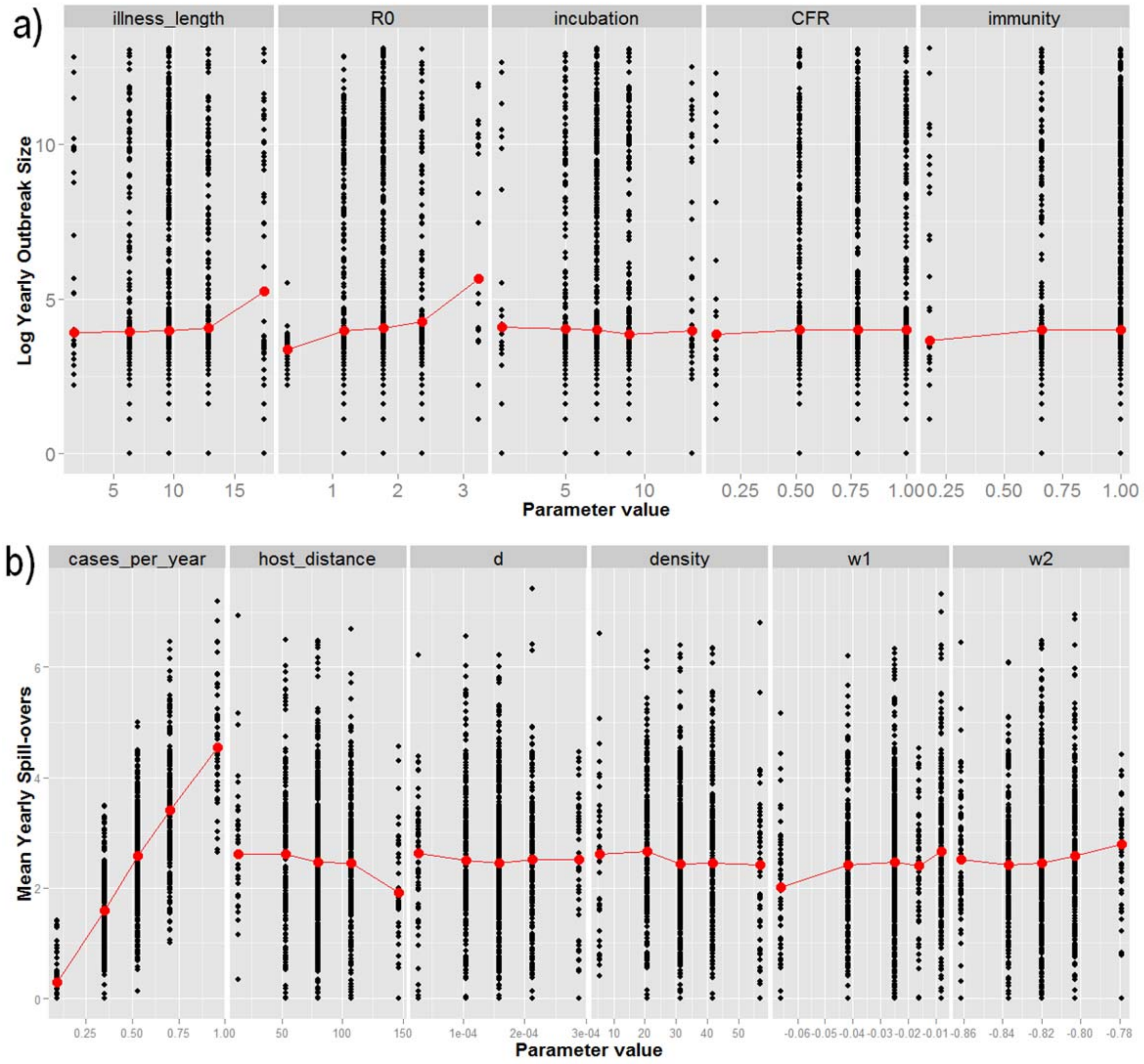

Supplementary Figure 5. Sensitivity plots of input parameters for (a) total number of annual log EVD cases, and (b) mean annual spill-overs. Black dots show the response values per simulation and are jittered for greater clarity. Red dots represent the median values for each parameter value, and red lines join the medians to aid interpretation of any trend. Parameters are as follows: illness length - mean number of days in the infectious compartment; R_0 – basic reproductive number; incubation - mean number of days in the exposed compartment; CFR - mean case fatality rate per illness; immunity - mean immunity to re-infection where 1 is totally immune; cases per year - mean spill-over rate constant; host distance - mean daily distance (m) travelled by host reservoir species; density - mean number of reservoir host individuals per grid cell; w2 - shape of the effective reproductive number (R_e) decay curve, where low values represent a less curved, more linear negative relationship; and w1 - shape of the CFR~poverty curve, where lower values represent a less curved, more linear negative relationship.

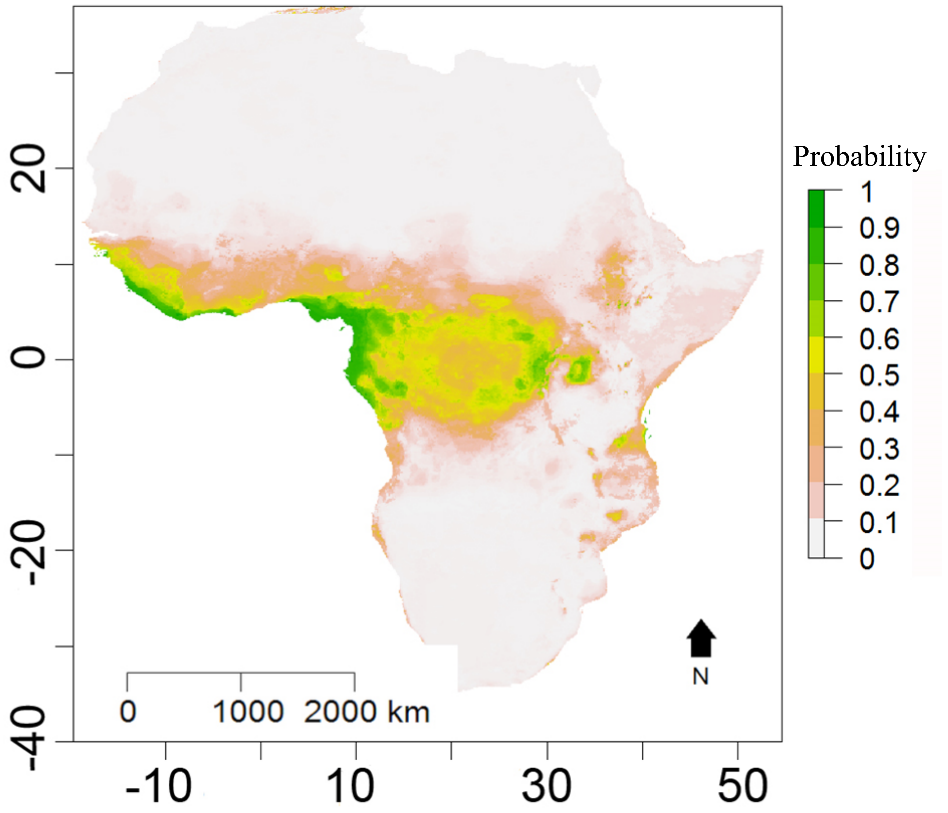

Supplementary Figure 6. **Future host occurrence probability.** Maps represents H_{2070} of EBOV host and other infection source species estimated from boosted-regression trees (BRT) models under the medium outlook RCP6 scenario. Probability of species occurrence per grid cell (0.0416°) is represented on a linear colour scale where green is most suitable (p(H) = 1) and white unsuitable (p(H) = 0) for all species combined. Axis labels indicate degrees in a World Geodetic System 84 projection.

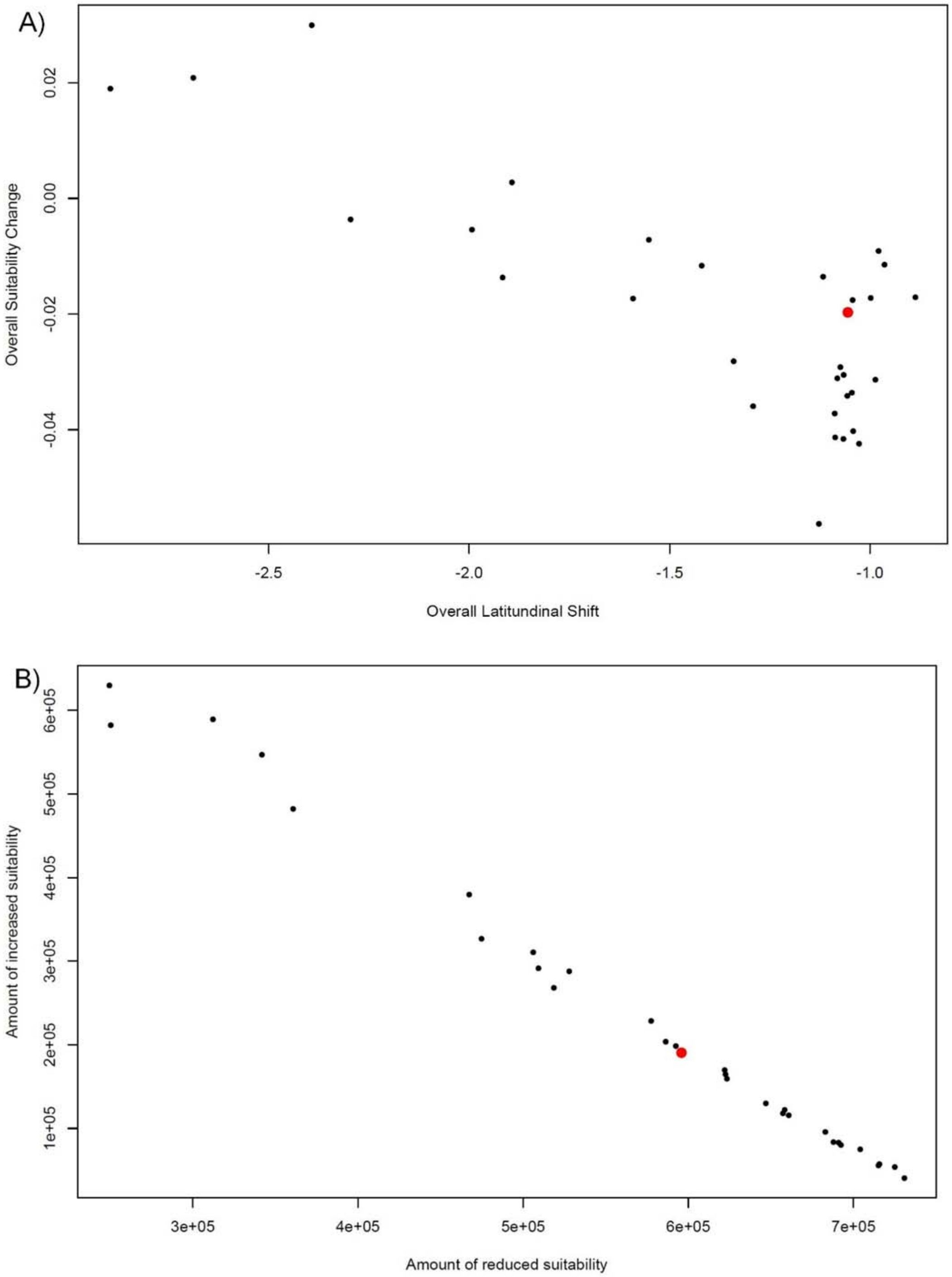

Supplementary Figure 7. Relative changes from present day to 2070 from host suitability grids using 32 different climate models. The host + non-host surfaces (see methods) were recreated using the same methods as for the present study but for all available climate models (n=32), with the climate model (HadGem2-AO) used in the present study highlighted in red. For panel (A) the points represent the mean change per grid cell in suitability from present day to 2070 versus the mean shift in non-zero points. For panel B the comparison is the number of grid cells that increase in value versus those that decrease in value.

Supplementary Tables

Supplementary Table 1. Seroprevalence of EBOV in reservoir host species. Species assignments followed the taxonomy in (10). Prevalence was measured as the proportion of positive results per sample and raw prevalence data was transformed to a rank within each study. Direct prevalence comparisons were not possible due to methodological differences. We estimated the most important EBOV host species as those that appear as the top two ranks in all sources. We identified four candidate bat species hosts: *Epomops franqueti*, *Epomophorus gambianus gambianus*, *Hypsignathus monstrosus*, and *Rousettus aegyptiacus*. N represents sample size; Hipp Hipposideridae; Molo Molossidae; Ptero Pteropodidae; CI Côte d'Ivoire; SL Sierra Leone; LR Liberia; GH Ghana; CG Congo; and GA Gabon.

Family	Species	Country	N	Prevalence	Rank	Source
Hipp	*Hipposideros sp.*	CG, SL, LR	98	0.04	4	(11)
Molo	*Mops condylurus*	CI, SL, LR, CG	37	0.05	4	(11)
Ptero	*Eidolon helvum*	GH	252	0.004	-	(12)
Ptero	*Epomophorus gambianus gambianus*	GH	37	0.38	2	(13)
Ptero	*Epomops franqueti*	GH	27	0.37	2	(13)
Ptero	*Epomops franqueti*	GA, CG	11	0.07	2	(14)
Ptero	*Epomops franqueti*	GA, CG	805	0.04	2	(15)
Ptero	*Epomops franqueti*	CI, SL, LR, CG	62	0.08	3	(11)
Ptero	*Hypsignathus monstrosus*	GH	16	0.44	1	(13)
Ptero	*Hypsignathus monstrosus*	GA, CG	17	0.24	1	(14)
Ptero	*Hypsignathus monstrosus*	GA, CG	125	0.07	1	(15)
Ptero	*Hypsignathus monstrosus*	CI, SL, LR, CG	70	0.16	2	(11)
Ptero	*Micropteropus pusillus*	GA, CG	197	0.02	4	(15)
Ptero	*Micropteropus sp.*	CG	40	0.03	4	(11)
Ptero	*Myonycteris torquata*	GA, CG	58	0.07	3	(14)
Ptero	*Myonycteris torquata*	GA, CG	573	0.03	3	(15)
Ptero	*Myonycteris torquata*	CI, SL, LR, CG	307	0.01	5	(11)
Ptero	*Nanonycteris veldkampii*	GH	4	0.25	3	(13)
Ptero	*Rousettus aegyptiacus*	GA, CG	307	0.08	1	(15)
Ptero	*Rousettus aegyptiacus*	CG	2	1.00	1	(11)

Supplementary Table 2. Details of bioclimatic and land use variables used to estimate probability of EBOV host presence, *H*. Nine most orthogonal (<75% correlation) bioclimatic variables were chosen from (16). For analysis, all variables were reduced in latitudinal extent to 85° N, 58° S and resampled to a 0.0416° grid cell size using a World Geodetic System 84 projection. LULC is a categorical dataset where the most predominant land use-land cover type in each grid cell is given within the following categories: Evergreen needle leaf forest; Evergreen broadleaf forest; Deciduous needle leaf forest; Deciduous broadleaf forest; Mixed forest; Closed shrublands; Open shrublands; Woody savannah; Grassland; Permanent wetlands; Cropland; Urban and built-up; Cropland/natural vegetation mosaic; Snow and ice; Barren or sparsely vegetated; and Water bodies.

No.	Variable Description	Original Spatial Extent	Original Spatial Resolution (cell size at equator)	Temporal Resolution	Source
1	BIO2 Mean Diurnal Temperature Range	Global	1km	2012	(16)
2	BIO5 Maximum Temperature of Warmest Month	Global	1km	2012	(16)
3	BIO6 Minimum Temperature of Coldest Month	Global	1km	2012	(16)
4	BIO7 Temperature Annual Range	Global	1km	2012	(16)
5	BIO10 Mean Temperature of Warmest Quarter	Global	1km	2012	(16)
6	BIO11 Mean Temperature of Coldest Quarter	Global	1km	2012	(16)
7	BIO12 Annual Precipitation	Global	1km	2012	(16)
8	BIO13 Precipitation of Wettest Month	Global	1km	2012	(16)
9	BIO14 Precipitation of Driest Month	Global	1km	2012	(16)
10	ALT Digital Elevation Model	Global	1km	2008	(17)
11	LULC Land Use-Land Cover	Global	500m	2001-2012	(18)

Supplementary Table 3. Estimates of global daily walking distances, $v\Delta t$. Estimates of daily walking distances were collected from the literature per country. Daily step numbers were converted to distance (km) using an average step length of 1.41m (19). As studies have suggested that daily walking distance is stratified among income categories (20), countries were assigned to income bands based on per capita Gross Domestic Product (GDP) (measured as Purchasing Power Parity from 2) such that the poorest countries were given a value of 1 and the richest 4. A mean estimate of walking distance was calculated for each band. Countries were then assigned a walking distance corresponding to their GDP band. No estimates were found for band 3 ($1600 - $35000), so countries in this band were given daily walking distances halfway between bands 2 and 4.

Country	Steps	Distance (km)	GDP band	GDP PPP Per capita (lower bound) $	GDP PPP Per capita (upper bound) $	Mean km Per GDP Band	Source
Niger	-	7	1	0	1600	9.6	(21)
Central African Republic	-	8	1	0	1600	9.6	(21)
Chad	-	15	1	0	1600	9.6	(21)
Mali	-	13.2	1	0	1600	9.6	(21)
Niger	-	4.8	1	0	1600	9.6	(21)
South Africa	12471	8.85	2	1600	13000	8.5	(22)
Tanzania	-	8.3	2	1600	13000	8.5	(23)
Australia	9695	6.88	4	35000	128530	5.6	(24)
Japan	7168	5.08	4	35000	128530	5.6	(24)
Switzerland	9650	6.85	4	35000	128530	5.6	(24)
United States	5117	3.63	4	35000	128530	5.6	(24)

Supplementary Table 4. Collated epidemiological data on EBOV outbreaks. Data on 19 locations that have experienced EBOV outbreaks or importations and have data on either Case Fatality Rate (CFR) (3, 25, 26, 27-31) or on Effective Reproductive Number change (5, 26, 27, 32-34) (R_e gradient per week). The latter data was either taken directly from tables or text from within literature sources or estimated (Spain, United Kingdom, Nigeria, United States) from descriptions of outbreak events detailed in the sources. Child mortality data for the year of outbreak is taken from World Bank Development Indicators (2)

Location	County	Year	ln GDP per capita for year	CFR	R_e gradient per week
United States	Texas	2014	4.74	0.3	0.5
Guinea		2014	3.09	0.707	0.113636
Sierra Leone		2014	3.31	0.69	0.076923
Liberia		2014	2.99	0.723	0.04
Germany		2014	4.66	0	
Spain	Madrid	2014	4.53	0	0.5
United Kingdom	London	2014	4.59	0	3
Nigeria		2014	3.77	0.666667	0.533333
Mali		2014	3.24	0.75	
Congo, Dem. Rep.		1976	2.72	0.88	0.105
Gabon		1994	4.14	0.61	
Congo, Dem. Rep.		1995	2.72	0.81	
Gabon		Early-1996	4.17	0.68	
Gabon		Late-1996	4.17	0.75	
Gabon		2001–2002	4.15	0.82	
Congo, Rep.		2001–2002	3.58	0.76	
Congo, Rep.		Early-2003	3.6	0.89	
Congo, Rep.		Late-2003	3.6	0.83	
Congo, Rep.		2005	3.65	0.75	

Supplementary Table 5. List of all 32 climate models used in analysis. List of all models (36) used to construct the host niche models with columns of name, origin and the ocean and atmosphere resolutions of the model.

Model Name	Institute and Country of Origin	Ocean horizontal resolution (°lat x °lon)	Atmosphere horizontal resolution (°lat x °lon)
ACCESS-1.0	CSIRO-BOM, Australia	1.0×1.0	1.9×1.2
ACCESS-1.3	CSIRO-BOM, Australia	1.0×1.0	1.9×1.2
CESM1-BGC	NSF-DOE-NCAR, USA	1.1×0.6	1.2×0.9
CESM1-CAM5	NSF-DOE-NCAR, USA	1.1×0.6	1.2×0.9
CanESM2	CCCMA, Canada	1.4×0.9	2.8×2.8
CCSM4	NCAR, USA	1.1×0.6	1.2×0.9
MRI-CGCM3	MRI, Japan	1.0×0.5	1.1×1.1
GFDL-CM3	NOAA, GFDL, USA	1.0×1.0	2.5×2.0
CanCM4	CCCMA, Canada	1.4×0.9	2.8×2.8
IPSL-CM5A-LR	IPSL, France	2.0×1.9	3.7×1.9
IPSL-CM5A-MR	IPSL, France	1.6×1.4	2.5×1.3
IPSL-CM5B-LR	IPSL, France	2.0×1.9	3.7×1.9
BCC-CSM1-1	BCC, CMA, China	1.0×1.0	2.8×2.8
BCC-CSM1-1-M	BCC, CMA, China	1.0×1.0	1.1×1.1
GISS-E2-H	NASA/GISS, NY, USA	2.5×2.0	2.5×2.0
GISS-E2-H-CC	NASA/GISS, NY, USA	1.0×1.0	1.0×1.0
GISS-E2-R	NASA/GISS, NY, USA	2.5×2.0	2.5×2.0
GISS-E2-R-CC	NASA/GISS, NY, USA	1.0×1.0	1.0×1.0
EC-EARTH	EC-EARTH, Europe	1.0×0.8	1.1×1.1
MIROC-ESM	JAMSTEC, Japan	1.4×0.9	2.8×2.8
MIROC-ESM-CHEM	JAMSTEC, Japan	1.4×0.9	2.8×2.8
MPI-ESM-LR	MPI-N, Germany	1.5×1.5	1.9×1.9
MPI-ESM-MR	MPI-N, Germany	0.4×0.4	1.9×1.9
GFDL-ESM2G	NOAA, GFDL, USA	1.0×1.0	2.5×2.0
GFDL-ESM2M	NOAA, GFDL, USA	1.0×1.0	2.5×2.0
FGOALS-g2	LASG, China	2.8 x 2.8	0.5x1
HadGEM2-AO	NIMR-KMA, Korea	1.0×1.0	1.9×1.2
HadGEM2-CC	MOHC, UK	1.0×1.0	1.9×1.2
HadGEM2-ES	MOHC, UK	1.0×1.0	1.9×1.2
MIROC5	JAMSTEC, Japan	1.6×1.4	1.4×1.4
CSIRO-Mk3-6-0	CSIRO-QCCCE, Australia	1.9×0.9	1.9×1.9
NorESM1-M	NCC, Norway	1.1×0.6	2.5×1.9

Supplementary References

1 Hutchinson, J. & Waser, P. M. Use, misuse and extensions of “ideal gas” models of animal encounter. Biological Reviews 3, 335-359 (2007).

2 World Bank. World Development Indicators. (World Bank, 2014).

3 Legrand, J., Grais, R. F., Boelle, P.-Y., Valleron, A.-J. & Flahault, A. Understanding the dynamics of Ebola epidemics. Epidemiology and Infection 135, 610-621 (2007).

4 Kiskowski, M. A. A three-scale network model for the early growth dynamics of 2014 west Africa ebola epidemic. PLoS currents 6 (2014).

5 Althaus, C. L., Low, N., Musa, E. O., Shuaib, F. & Gsteiger, S. Ebola virus disease outbreak in Nigeria: transmission dynamics and rapid control. Epidemics 11, 80-84 (2015).

6 Nishiura, H. & Chowell, G. Early transmission dynamics of Ebola virus disease (EVD), West Africa, March to August 2014. Eurosurveillance 19, 20894, doi:10.2807/1560-7917.ES2014.19.36.20894 (2014).

7 Elvidge, C. D. et al. A global poverty map derived from satellite data. Computers & Geosciences 35, 1652-1660 (2009).

8 Global Biodiversity Information Facility. (http://www.gbif.org/, 2013).

9 Pigott, D. M. et al. Mapping the zoonotic niche of Ebola virus disease in Africa. Elife 3, e04395 (2014).

10 Wilson, D. E. & Reeder, D. M. Vol. 1 (John Hopkins University Press, Baltimore, 2005).

11 Weiß aus Karlsruhe, S. Identification and characterisation of emerging viruses in free-ranging bats from sub-Saharan Africa EngD Thesis thesis, Technical University of Berlin, (2013).

12 Hayman, D. T. S. et al. Long-Term Survival of an Urban Fruit Bat Seropositive for Ebola and Lagos Bat Viruses. PloS one 5, e11978 (2010).

13 Hayman, D. T. S. et al. Ebola virus antibodies in fruit bats, ghana, west Africa. Emerging infectious diseases 18, 1207-1209, doi:10.3201/eid1807.111654 (2012).

14 Leroy, E. M. et al. Fruit bats as reservoirs of Ebola virus. Nature 438, 575-576, doi:10.1038/438575a (2005).

15 Pourrut, X. et al. Large serological survey showing cocirculation of Ebola and Marburg viruses in Gabonese bat populations, and a high seroprevalence of both viruses in Rousettus aegyptiacus. BMC infectious diseases 9, 159, doi:10.1186/1471-2334-9-159 (2009).

16 Hijmans, R. J., Cameron, S. E., Parra, J. L., Jones, P. G. & Jarvis, A. Very high resolution interpolated climate surfaces for global land areas. International Journal of Climatology 25, 1965-1978 (2005).

17 Jarvis, A., Reuter, H. I., Nelson, A. & Guevara, E. (CGIAR-CSI SRTM 90m Database, 2008).

18 Friedl, M. A. et al. MODIS Collection 5 global land cover: Algorithm refinements and characterisation of new datasets. Remote Sensing of the Environment 114, 168-182 (2010).

19 Barreira, T. V., Rowe, D. A. & Kang, M. Original Research Parameters of Walking and Jogging in Healthy Young Adults. International Journal of Exercise Science 3, 4-13 (2010).

20 Hallal, P. C. et al. Global physical activity levels: surveillance progress, pitfalls, and prospects. The Lancet 380, 247-257 (2012).

21 Filmer, D. If You Build It, Will They Come? School Availability and School Enrollment in 21 Poor Countries. (The World Bank, 2004).

22 Cook, I., Alberts, M. & Lambert, E. V. Relationship between adiposity and pedometer-assessed ambulatory activity in adult, rural African women. International Journal of Obesity 32, 1327-1330 (2008).

23 Marlowe, F. The Hadza: Hunter-gatherers of Tanzania. (University of California Press, 2010).

24 BASSETT, D. R. J., WYATT, H. R., THOMPSON, H., PETERS, J. C. & HILL, J. O. Pedometer-Measured Physical Activity and Health Behaviors in U.S. Adults. Medicine & Science in Sports & Exercise 42, 1819-1825, doi:10.1249/MSS.0b013e3181dc2e54 (2010).

25 Chowell, G., Hengartner, N. W., Castillo-Chavez, C., Fenimore, P. W. & Hyman, J. M. The basic reproductive number of Ebola and the effects of public health measures: The cases of Congo and Uganda. Journal of Theoretical Biology 229, 119-126, doi:10.1016/j.jtbi.2004.03.006 (2004).

26 Althaus, C. L. Estimating the reproduction number of Ebola virus (EBOV) during the 2014 outbreak in West Africa. arXiv preprint arXiv:1408.3505 (2014).

27 Chowell, G. & Nishiura, H. Transmission dynamics and control of Ebola virus disease (EVD): a review. BMC medicine 12, 196 (2014).

28 Lefebvre, A. et al. Case fatality rates of Ebola virus diseases: a meta-analysis of World Health Organization data. Médecine et maladies infectieuses 44, 412-416 (2014).

29 Kucharski, A. J. & Edmunds, W. J. Case fatality rate for Ebola virus disease in west Africa. The Lancet 384, 1260 (2014).

30 Fasina, F. O. et al. Transmission dynamics and control of Ebola virus disease outbreak in Nigeria, July to September 2014. Euro Surveill 19, 20920 (2014).

31 Rouquet, P. et al. Wild animal mortality monitoring and human Ebola outbreaks, Gabon and Republic of Congo, 2001–2003. Emerging infectious diseases 11, 283 (2005).

32 Team, W. E. R. Ebola virus disease in West Africa—the first 9 months of the epidemic and forward projections. N Engl J Med 2014, 1481-1495 (2014).

33 Towers, S., Patterson-Lomba, O. & Castillo-Chavez, C. Temporal variations in the effective reproduction number of the 2014 West Africa Ebola outbreak. PLoS currents 6 (2014).

34 Althaus, C. L. Estimating the reproduction number of Ebola virus (EBOV) during the 2014 outbreak in West Africa. PLoS currents 6 (2014).

35 European Network for Earth Systems Modelling: CMIP5 Models and Grid Resolution (https://portal.enes.org/data/enes-model-data/cmip5/resolution, 2018).
